# Cardiac structure and function during ageing in energetically compromised Guanidinoacetate N-methyltransferase (GAMT)-knockout mice – a one year longitudinal MRI study

**DOI:** 10.1186/1532-429X-10-9

**Published:** 2008-02-06

**Authors:** Jürgen E Schneider, Lee-Anne Stork, Jordana T Bell, Michiel ten Hove, Dirk Isbrandt, Kieran Clarke, Hugh Watkins, Craig A Lygate, Stefan Neubauer

**Affiliations:** 1 Department of Cardiovascular Medicine, University of Oxford, Oxford, UK; 2 Wellcome Trust Centre for Human Genetics, University of Oxford, Oxford, UK; 3 Department of Physiology, Anatomy and Genetics, University of Oxford, Oxford, UK; 4 Centre for Molecular Neurobiology Hamburg (ZMNH), Institute for Neural Signal Transduction, Hamburg, Germany

## Abstract

**Background:**

High-resolution magnetic resonance imaging (cine-MRI) is well suited for determining global cardiac function longitudinally in genetically or surgically manipulated mice, but in practice it is seldom used to its full potential. In this study, male and female guanidinoacetate N-methyltransferase (GAMT) knockout, and wild type littermate mice were subjected to a longitudinal cine-MRI study at four time points over the course of one year. GAMT is an essential enzyme in creatine biosynthesis, such that GAMT deficient mice are entirely creatine-free. Since creatine plays an important role in the buffering and transfer of high-energy phosphate bonds in the heart, it was hypothesized that lack of creatine would be detrimental for resting cardiac performance during ageing.

**Methods:**

Measurements of cardiac structure (left ventricular mass and volumes) and function (ejection fraction, stroke volume, cardiac output) were obtained using high-resolution cine-MRI at 9.4 T under isoflurane anaesthesia.

**Results:**

There were no physiologically significant differences in cardiac function between wild type and GAMT knockout mice at any time point for male or female groups, or for both combined (for example ejection fraction: 6 weeks (KO vs. WT): 70 ± 6% vs. 65 ± 7%; 4 months: 70 ± 6% vs. 62 ± 8%; 8 months: 62 ± 11% vs. 62 ± 6%; 12 months: 61 ± 7% vs. 59 ± 11%, respectively).

**Conclusion:**

These findings suggest the presence of comprehensive adaptations in the knockout mice that can compensate for a lack of creatine. Furthermore, this study clearly demonstrates the power of cine-MRI for accurate non-invasive, serial cardiac measurements. Cardiac growth curves could easily be defined for each group, in the same set of animals for all time points, providing improved statistical power, and substantially reducing the number of mice required to conduct such a study. This technique should be eminently useful for following changes of cardiac structure and function during ageing.

## Introduction

Magnetic resonance imaging (cine-MRI) is 3D-capable, non-invasive, with high spatial resolution, and represents the most sophisticated tool to determine cardiac structure and function in normal, genetically or surgically manipulated mice [1-4]. Hearts of juvenile or adult transgenic mouse models are commonly examined and compared to wild type littermates at a single time point to identify and characterize the effect of the genetic alteration(s) on global cardiac performance. However, due to the non-invasiveness of MRI, mice can be investigated in a longitudinal fashion in order to follow-up after an intervention such as myocardial infarction [5], transverse aortic constriction [6], or to identify phenotypes that may occur as a function of ageing.

Here we report on the longitudinal application of cine-MRI on a mouse model of guanidinoacetate N-methyltransferase (GAMT)-deficiency [7]. GAMT catalyses the second essential step in creatine synthesis, and consequently hearts from these mice (when fed a creatine free diet) completely lack creatine and phosphocreatine (PCr), as we have previously confirmed non-invasively using ^1^H-MRS and using HPLC [8]. Creatine is thought to play an important role in cardiomyocytes contributing to the creatine-kinase system, which is both an energy buffer and a transport system shuttling high-energy phosphate bonds (in the form of PCr) from the mitochondria to the myofibrils [9]. Indeed a loss of myocardial creatine has commonly been associated with the development of heart failure [10], and reducing LV creatine concentration prior to coronary artery ligation renders rats unable to survive a myocardial infarction [11].

Our earlier studies in younger GAMT ko mice have demonstrated only a small decrease in left ventricular (LV) systolic pressure at rest, but a pronounced reduction in contractile reserve in response to β-adrenergic receptor stimulation [12]. However, this phenotype may represent only the start of a longer progressive deterioration. For example, mice over-expressing the β_2_-adrenergic receptor have no evidence of cardiac dysfunction at 4 months, yet go on to develop overt heart failure by 12 months of age [13]. Therefore, in the present study, we investigated the hypothesis that lack of creatine would be detrimental for resting cardiac performance during ageing. To this purpose we subjected male and female wild type and GAMT-ko mice to a longitudinal cine-MRI study over a time period of one year.

## Materials and Methods

### Animal preparation

All mice were backcrossed on to a C57Bl/6J background for at least 8 generations. Knockout and wild type mice were genotyped by polymerase chain reaction (PCR) methods, and housed separately according to genotype to prevent accumulation of creatine via coprophagia of faeces from wild type animals. All investigations conform to UK Home Office *Guidance on the Operation of the Animals (Scientific Procedures) Act*, 1986 (HMSO) and to institutional guidelines.

Male and female GAMT ko and wild type littermate mice (*n *= 7 per group and sex) were kept in cages with 12 h light-dark cycle and controlled temperature (20–22°C), and fed creatine free chow and water *ad libitum*. Cine-MRI studies were performed at the age of 6 weeks, 4, 8 and 12 months. After inducing anesthesia in an anesthetic chamber using 4% isoflurane in 100% oxygen, animals were positioned supine in a purpose-built animal holder for positioning mice vertically, and maintained at 1.5–2% isoflurane in 1 l/min oxygen flow throughout the MR experiments. Cardiac and respiratory signals were continuously monitored using an in-house developed ECG- and respiratory gating device [14]. Both signals were derived from two electrodes inserted subcutaneously in the front paws. Respiratory signals could also be obtained from a loop loosely fitted to the chest and abdomen of the animals. Temperature was maintained using a blanket that was heated by warm air. Mice were secured within the holder using surgical tape, without compressing their abdomen or chest regions.

### Magnetic Resonance Imaging

MR experiments were carried out on an 11.7 T (500 MHz) MR system comprising a vertical magnet (bore size 123 mm – Magnex Scientific, Oxon, UK), a Bruker Avance console (Bruker Medical, Ettlingen, Germany) and a shielded gradient system (548 mT/m, 160 μs rise time) (Magnex Scientific, Oxon, UK). Quadrature driven birdcage coils with inner diameters of 28 mm and 40 mm (Rapid Biomedical, Würzburg, Germany) were used according to the body weight of the animal. High-resolution cine-MRI was performed as described previously, using a fast gradient echo sequence [15]. In brief, seven to ten contiguous slices (slice thickness 1 mm) were acquired in short-axis orientation covering the entire heart. The imaging parameters were: field-of-view (25.6 mm)^2^, matrix size 256 × 256, echo time/repetition time = 1.43/4.6 ms, α = 15°, number of averages = 2. The sequence was ECG-triggered and respiratory gated, the total scan-time per animal ranged from 30 to 60 mins. 20–30 frames per cardiac cycle were acquired depending on the heart rate.

### Data analysis

Image reconstruction and data reconstruction was performed off-line, using purpose-written *idl*-software (Research Systems International, Crowthorne, Berkshire, UK). Raw data were isotropically zerofilled by a factor of two and filtered prior to Fourier transformation resulting in an in-plane voxel size of 50 × 50 μm and then exported into TIFF-format. For segmentation, the TIFF-images were loaded into Amira™ 2.3 (TGS Europe, Mérignac Cedex, France). End-diastolic and end-systolic frames were selected according to maximal and minimal ventricular volume. Based on end-systolic (ESV) and end-diastolic (EDV) volumes, all parameters characterising cardiac function, such as stroke volume (SV = EDV-ESV), ejection fraction (EF = SV/EDV) and cardiac output (CO = SV × heart rate), were calculated. Furthermore, LV volumes of a mid-ventricular slice were segmented in all cine-frames, normalized to the EDV of this slice to control for differences in chamber size, and subjected to a Fourier analysis [16,17] using four harmonics in order to obtain maximum rates of volume change as a measure of contraction and relaxation i.e. (dV/dt)_min/max_·EDV^-1^. All values are given as mean ± SD. The data from each trait were statistically analyzed using a linear mixed-effects model with mouse number (n = 28) as random factor, and fixed factors specified in the following order: gender (n = 2), genotype (n = 2), and time (n = 4). Because genotype had a significant effect on body weight, in the analysis of the five structural parameters in this study (LVM, EDV, ESV, SV, and CO) body weight was also included as a factor in the model. The fit of the full model, which included the main effects and all pair-wise fixed interaction terms, was assessed first, and then the non-significant interaction terms were dropped from the model. The fit of the main-effects-only model was also assessed. We present P-values uncorrected for multiple comparisons, where a value of P < 0.01 was considered significant.

## Results

In general, MR examinations were well tolerated with few adverse effects. No significant difference in mortality was observed; with a total of 3 KO mice (2 male + 1 female) and 1 WT (female) mouse dying over the one year time course of this study. In all cases, mice either failed to make a full recovery from general anesthesia or died within a few days after the 8 month MR-exam.

**Figure 1:**
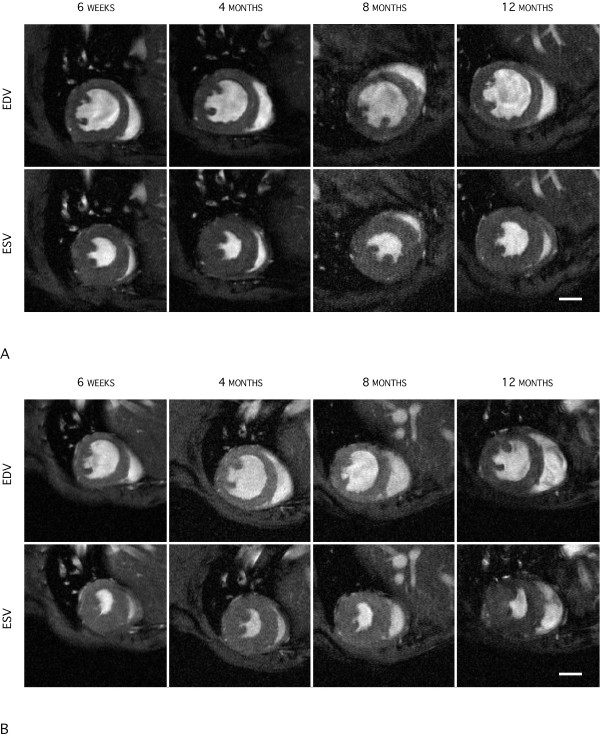
(a) Mid-ventricular end-diastolic (top row) and end-systolic (bottom row) frames in the short-axis orientation of a male wild type mouse heart for the four different time points covering the study duration of one year. (b) Corresponding mid-ventricular end-diastolic (top row) and end-systolic (bottom row) frames in the short-axis orientation of a male GAMT-ko mouse heart at the respective time points. While the hearts of the transgenic mice were significantly smaller, cardiac function did not deteriorate over time, despite the lack of creatine. Scale bars: 2 mm.

**Figure 2:**
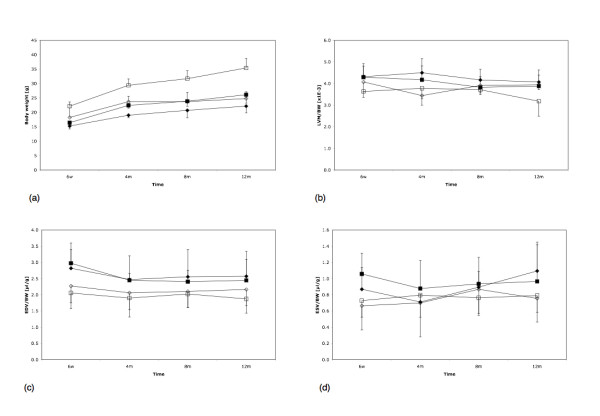
(a) Bodyweight (BW), (b) left ventricular mass (LVM), (c) EDV, and (d) ESV for all four groups (open square – male wt; open diamond – male ko; black diamond – female ko; black square – female wt) as a function of time. LVM, EDV and ESV were normalized to the respective body weight.

Figure 1a shows representative end-diastolic (top row) and end-systolic (bottom row) frames of a male WT at 6 weeks, 4, 8 and at 12 months (from left to right). Figure 1b shows the corresponding frames of a male GAMT-ko mouse. Both figures underline the image quality obtainable in such a longitudinal study. GAMT-ko mice had significantly lower body weight compared to wild type controls at all time points in males, and from 4 months of age in females (Figure 2a). For this reason, all structural parameters have been presented normalized to body weight. However, mean values before normalization for all cardiac structural and functional parameters are listed in Table 1.

**Table 1:** Cardiac Parameters for Male and Female WT and GAMT-ko Mice measured by MRI

		6 weeks		4 months		8 months		12 months	
		WT	GAMT-ko	WT	GAMT-ko	WT	GAMT-ko	WT	GAMT-ko

*n*	M **F**	7 **7**	7 **7**	7 **7**	7 **7**	7 **7**	7 **7**	7 **6**	5 **6**
Body weight (*g)*	M **F**	22 ± 2 **16 ± 1**	17 ± 3 **15 ± 1**	29 ± 3 **22 ± 1**	24 ± 2 **19 ± 1**	32 ± 3 **24 ± 2**	24 ± 3 **21 ± 3**	35 ± 3 **26 ± 3**	25 ± 2 **22 ± 2**
LV Mass *(mg)*	M **F**	80 ± 6 **71 ± 11**	70 ± 13 **67 ± 9**	108 ± 12 **94 ± 23**	82 ± 11 **86 ± 9**	118 ± 9 **91 ± 14**	92 ± 11 **86 ± 8**	112 ± 19 **101 ± 14**	98 ± 8 **90 ± 9**
End-diastolic volume *(μl)*	M **F**	45 ± 6 **49 ± 10**	39 ± 12 **44 ± 9**	54 ± 8 **55 ± 18**	49 ± 18 **47 ± 4**	64 ± 13 **58 ± 9**	50 ± 16 **52 ± 15**	66 ± 12 **63 ± 20**	54 ± 15 **57 ± 11**
End-systolic volume *(μl)*	M **F**	16 ± 5 **17 ± 5**	11 ± 5 **13 ± 4**	23 ± 8 **20 ± 9**	16 ± 10 **14 ± 1**	24 ± 7 **22 ± 4**	21 ± 10 **18 ± 7**	28 ± 7 **25 ± 12**	19 ± 9 **24 ± 7**
Stroke volume *(μl)*	M **F**	29 ± 4 **31 ± 6**	28 ± 8 **30 ± 6**	31 ± 5 **35 ± 10**	32 ± 8 **34 ± 4**	40 ± 9 **35 ± 8**	29 ± 9 **34 ± 10**	38 ± 13 **39 ± 9**	35 ± 8 **33 ± 5**
Ejection fraction *(%)*	M **F**	65 ± 7 **64 ± 4**	72 ± 4 **70 ± 7**	58 ± 12 **65 ± 4**	68 ± 7 **71 ± 4**	62 ± 7 **61 ± 6**	58 ± 13 **65 ± 8**	56 ± 14 **62 ± 6**	66 ± 6 **58 ± 6**
Heart rate *(bpm)*	M **F**	485 ± 42 **485 ± 25**	506 ± 20 **483 ± 46**	555 ± 63 **472 ± 27**	465 ± 35 **496 ± 45**	514 ± 25 **421 ± 24**	450 ± 31 **479 ± 43**	488 ± 46 **451 ± 51**	441 ± 66 **497 ± 14**
Cardic output *(ml · min*^-1^*)*	M **F**	14.2 ± 1.4 **15.2 ± 3.1**	13.9 ± 3.3 **14.4 ± 2.8**	17.3 ± 3.3 **16.6 ± 4.7**	14.9 ± 3.4 **14.6 ± 6.6**	20.3 ± 4.0 **14.8 ± 3.4**	13.1 ± 4.5 **16.0 ± 3.9**	18.1 ± 6.0 **17.3 ± 3.8**	15.9 ± 5.5 **16.1 ± 2.3**
dV/dt_max_.EDV^-1^ *(10*^3^·*s*^-1^*)*	M **F**	20.5 ± 5.0 **24.7 ± 1.7**	21.6 ± 3.5 **23.2 ± 4.7**	21.5 ± 5.0 **22.1 ± 4.3**	19.3 ± 1.7 **23.3 ± 3.9**	17.8 ± 3.2 **19.9 ± 2.9**	18.0 ± 2.1 **21.3 ± 2.0**	17.8 ± 3.7 **18.7 ± 3.5**	20.2 ± 4.1 **20.5 ± 1.3**
dV/dt_min_.EDV^-1^ *(10*^3^· *s*^-1^*)*	M **F**	-18.2 ± 3.9 **-15.0 ± 1.6**	-21.3 ± 4.7 **-18.6 ± 3.9**	-17.8 ± 1.5 **-16.7 ± 4.2**	-21.7 ± 5.5 **-20.8 ± 2.3**	-19.4 ± 4.6 **-15.0 ± 3.9**	-16.7 ± 3.6 **-17.3 ± 3.3**	-15.8 ± 3.0 **-16.8 ± 5.1**	-17.5 ± 4.5 **-15.8 ± 3.3**

All parameters are for the left ventricle(LV) and represent mean ± standard deviation.

Each of the ten traits (i.e. BW, LVM, EDV, ESV, SV, EF, CO, HR, (dV/dt)_max_·EDV^-1 ^and (dV/dt)_min_·EDV^-1^, respectively) were analyzed using a linear mixed-effects model, which assessed the effect of gender, genotype, time, and their interactions on the phenotype. In addition, for five of the traits (LVM, EDV, ESV, SV, and CO) we also controlled for the effect of body weight on the parameter, by including BW as a factor in the model. For each trait we initially obtained the fit of the full model, and then dropped the interaction terms that were not significant. The final refitted model included all main effects terms and the significant interactions, which are listed in Table 2. The residuals versus the fitted responses from the model were plotted for each response variable, and no substantial deviations from the assumptions of constant variance of the residuals were observed. The estimates of the effect sizes and significance values obtained (Table 2) for the main effects were not substantially affected when main-effects-only models were fitted to the data, or by the order of the variables in the model for main-effects-only models. Linear regression of these data with robust variance estimation by clustering on mouse identifier yielded comparable results (data not shown).

**Table 2:** Summary of the significant findings from the mixed-effects regression

**Trait ^a^**	**Factor ^b^**	**Coefficient estimate**	**95% CI**	***P*-value**
BW	Sex	-5.13	[-6.72, -3.54]	<0.0001
	Genotype	-2.40	[-4.3, -0.49]	<0.0001
	Time	-	-	<0.0001
	4 months	6.34	[4.86, 7.82]	<0.0001
	8 months	8.28	[6.9, 9.66]	<0.0001
	12 months	11.73	[10.33, 13.13]	<0.0001
	Genotype*Time	-	-	0.0003
	Genotype*4 months	-1.52	[-3.55, 0.52]	0.1415
	Genotype*8 months	-2.60	[-4.56, -0.64]	0.0102
	Genotype*12 months	-4.79	[-6.88, -2.69]	<0.0001
LVM	BW	0.88	[-0.06, 1.81]	<0.0001
	Sex	-4.50	[-13.36, 4.35]	0.2636
	Genotype	-8.85	[-16.91, -0.78]	0.7443
	Time	-	-	0.0003
	4 months	15.35	[7.9, 22.8]	0.0001
	8 months	18.55	[10.27, 26.84]	<0.0001
	12 months	19.29	[8.84, 29.74]	0.0005
EF	Sex	1.06	[-2.55, 4.66]	0.4924
	Genotype	3.46	[-0.02, 6.94]	0.0334
	Time	-	-	0.0027
	4 months	-1.43	[-5.58, 2.72]	0.4947
	8 months	-5.42	[-9.44, -1.4]	0.009
	12 months	-7.30	[-11.55, -3.06]	0.001
Heart rate	Sex	-48.49	[-74.64, -22.33]	0.1958
	Genotype	-42.08	[-67.96, -16.2]	0.698
	Time	-	-	0.0519
	4 months	4.99	[-17.61, 27.58]	0.6611
	8 months	-23.16	[-44.79, -1.54]	0.0362
	12 months	-20.67	[-43.49, 2.15]	0.0751
	Sex*Genotype	73.26	[35.93, 110.6]	0.0004
dV/dt_max_.EDV^-1^	Sex	0.002	[0.001, 0.004]	0.0048
	Genotype	0.000	[-0.001, 0.002]	0.8454
	Time			0.0009
	4 months	-0.001	[-0.003, 0.001]	0.2688
	8 months	-0.003	[-0.005, -0.001]	0.0017
	12 months	-0.004	[-0.005, -0.002]	0.0004

^a ^Traits for which at least one factor in the model had significant (P < 0.01) effects.

^b ^Estimated effects of KO compared to WT (baseline) for Genotype, female compared to male (baseline) for Sex, and trait values at 4 months, 8 months, and 12 months compared to trait values at 6 weeks (baseline) for Time (3df test)

All factors had significant main effects for body weight as mentioned above. Furthermore, a significant (P < 0.01) interaction was obtained for body weight between between genotype and time (F = 7.3, df = [3,68], P = 0.0003), indicating a different growth curve between KO and WT mice, i.e. WT had a higher body weight compared to KO at all time points (Tables 1 and 2).

Gender has a significant effect on body weight and on dV/dtmax .EDV^-1 ^(P < 0.0001 and P = 0.0048, respectively). The majority of the traits showed significant variation across time (Table 2). However, no significant interaction between genotype and time was found for LVM or EDV, indicating the absence of progressive LV dilatation or LV hypertrophy.

**Figure 3:**
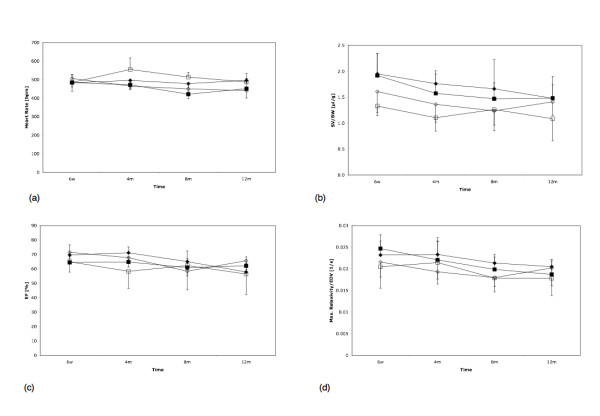
(a) Heart rate (HR), (b) stroke volume (SV) (c) ejection fraction (EF), and (d) (dV/dt)_max_·EDV^-1 ^for all four groups (open square – male wt; open diamond – male ko; black diamond – female ko; black square – female wt) as a function of time. SV was normalized to the respective body weight, the time-volume curves to EDV before fitting.

Significant interactions were also obtained between gender and genotype for heart rate (F = 16.33, df = [1,25], P = 0.0004), i.e. female WTs had lower heart rate than male WTs at most time points; but the converse of this was true for KO mice. The significance of this is unclear, especially as all heart rates were typically > 450 bpm for all groups and at all time points indicating that physiological conditions under isoflurane anaesthesia were stable and reproducible during the MR-examinations (see also Fig. 3a).

LV functional parameters such as EDV, ESV, stroke volume, and EF were not different between KO and WT mice, and remained within the normal range at all time points (Fig. 2c, d and Fig. 3b, c). There was a small but significant trend towards an age-related decline in systolic function in all experimental groups (EF, P = 0.0027). Maximum rates of contraction and relaxation were calculated as the maximum and minimum rate of volume change. Since this parameter is sensitive to differences in LV chamber size, all volumes were normalized to EDV prior to fitting. No differences were observed between genotypes, or over time, for minimum rate of volume change (dV/dt)_min_·EDV^-1 ^(Table 1). The maximum rate of volume change (dV/dt)_max_·EDV^-1 ^as a measure of relaxation showed a small decrease over time (Fig. 3d; P = 0.0009). Moreover, female mice tended to have a higher (dV/dt)_max_·EDV^-1 ^(P = 0.0048).

## Discussion

We have used high-resolution cine-MRI to systematically assess cardiac function in a transgenic, creatine free, mouse model of GAMT deficiency [7,8,18]. We applied this technique repeatedly in the same animals covering a time period of 12 months to address the question whether cardiac function in these mice would deteriorate with age. Creatine plays a crucial role in the energy metabolism of the heart as a buffer and a carrier of high-energy phosphates [9]. A loss of creatine is characteristic for the failing heart, and has been postulated as one major mechanism leading to contractile dysfunction due to energetic derangement [19]. It is therefore surprising that this study did not reveal any evidence for LV hypertrophy or contractile dysfunction under baseline conditions in the creatine-deficient GAMT ko mice even, at the age of 12 months.

GAMT ko mice have a combination of altered body composition and growth making meaningful comparison of LV mass and volumes between genotypes difficult. Body weight is up to 30% lower mainly due to a reduced total body fat content [7], while long bone length is reduced by ~5% [12]. However, we have previously shown that molecular markers of cardiac hypertrophy are not significantly elevated in GAMT mice at 5 months of age confirming that these mice do not have LV hypertrophy [12]. Since in the present study, LVmass to body weight ratio did not change with time compared to WT animals (Figure 2b), we now conclude that LV hypertrophy does not develop during ageing in the GAMT ko mice. A possible refinement to the current study would be to non-invasively measure another parameter that more accurately reflects body size when body composition is altered e.g. tibial length or brain volume. However, this would add significantly to the time taken for the imaging protocol, especially as these areas are outside our RF-coils used for cardiac imaging. While female mice tended to have a statistically significant altered relaxation compared to the male animals, it seems unlikely that these relatively small differences are physiologically relevant.

One limitation of this study is that, in order to keep the protocol as non-invasive as possible and to avoid loss of animals during the imaging procedure, we did not make acute measurements of cardiac functional reserve, which we have previously shown to be impaired in GAMT ko mice [12]. This requires the parenteral administration of a β-adrenergic agonist, preferably intravenously (IV). However, IV access in the mouse is not readily obtainable for dosing at multiple time points, while drug absorption from intraperitoneal injection is highly variable resulting in poor repeatability.

Three GAMT ko mice and one WT died after being subjected to general anesthesia at the 8 month imaging time point. Although more mice died in the GAMT ko group, this was not statistically significant, nor was this study powered to determine differences in mortality. It is likely that these deaths were associated with a general increased risk from exposure to general anaesthesia with age. The death of the three GAMT ko mice resulted in an unbalanced data design in the statistical analysis. However, similar significance findings were obtained when re-analyzing the data with a balanced design (i.e. completely dropping out the individuals with missing data points – data not shown).

In the current study we were interested in detecting physiologically relevant differences according to sex and genotype by imaging the same sample of mice serially, rather than collecting a larger sample of mice with non-serial measurements. The study design therefore incorporated correlated measures across different time points in development. Mixed effects models provide a powerful tool for the analysis of such grouped data. The increased power of this approach allows for a reduced sample size needed for this study. In addition, our sample has satisfactory power to detect statistically significant differences of physiological importance. For example, the power to detect a physiologically significant difference in EF of at least 10 units is greater than 95% if analyzing the sample at a single time point using a one-sample two-sided *t*-test at a significance level of 0.01 to correct for multiple comparisons (assuming a change in the mean EF of 10 units, the observed standard deviation of EF of 8.42 units in the entire sample, 26 animals per group since 2 animals were lost from the male KO group, and an alpha level of 0.01).

The ability of GAMT ko mice to maintain normal cardiac function in the complete absence of phosphocreatine is remarkable. A major factor in this is probably the accumulation of the creatine precursor guanidinoacetate, which can participate in the creatine kinase reaction albeit at 1% of the reaction velocity for phosphocreatine [20]. Since the GAMT knockout is not time-specific, other adaptations are also likely, and major compensatory mechanisms may develop during embryonic development. Further experiments are ongoing to investigate such potential adaptation in more detail.

The non-invasive, longitudinal nature of MRI is often discussed but seldom utilized to its full potential. In the current climate of improving animal welfare, there is an ethical imperative to implement the principles of replacement, reduction and refinement within the context of animal experimentation. In the current study, we were able to image the same mice serially, such that a total of 28 mice were used in the entire study, rather than the more than 112 animals that would be required for non-serial measurements. An equivalent reduction could also have been achieved using echocardiography; however the superior spatial resolution of MRI also enables fewer animals to be used per group compared to echocardiography. For example, we used 7 animals per genotype and sex, compared to 12 animals per group for a similar longitudinal study using echocardiography in mice [13]. It should also be noted that 3-D volume measurements in mice using echocardiography has only recently been described, and has yet to become standard laboratory practice [21].

In conclusion, this study shows that GAMT ko mice have normal cardiac structure and function at rest, which remains normal during ageing. Furthermore, to the best of our knowledge this is the first MR-study to report on the longitudinal investigation of a transgenic mouse model over the period of one year, demonstrating the power of the MR-technique to accurately quantify cardiac functional parameters in genetically modified mice in a longitudinal fashion. Importantly, each animal served as its own control, providing a more powerful statistical analysis and substantially reducing the number of mice required to conduct such a study.
